# Effects of 12 mg vs. 6 mg dexamethasone on thromboembolism and bleeding in patients with critical COVID-19 - a post hoc analysis of the randomized, blinded COVID STEROID 2 trial

**DOI:** 10.1186/s13613-023-01115-y

**Published:** 2023-03-02

**Authors:** Sandra Jonmarker, Felix Alarcón, Jacob Litorell, Anders Granholm, Eva Joelsson Alm, Michelle Chew, Lene Russell, Sarah Weihe, Emilie Kabel Madsen, Nick Meier, Jens Wolfgang Leistner, Johan Mårtensson, Jacob Hollenberg, Anders Perner, Maj-Brit Nørregaard Kjær, Marie Warrer Munch, Martin Dahlberg, Maria Cronhjort, Rebecka Rubenson Wahlin

**Affiliations:** 1 Department of Clinical Science and Education, Karolinska Institutet, Södersjukhuset, Stockholm, Sweden; 2 Department of Anaesthesia and Intensive Care, Södersjukhuset, Sjukhusbacken 10, SE-118 83 Stockholm, Sweden; 3 Department of Intensive Care, Rigshospitalet, Copenhagen University Hospital, Copenhagen, Denmark; 4 Collaborations for Research in Intensive Care (CRIC), Copenhagen, Denmark; 5 Department of Anaesthesia and Intensive Care Medicine, Biomedical and Clinical Sciences, Linköping University, Linköping, Sweden; 6 Department of Anaesthesiology and Intensive Care, Copenhagen University Hospital Gentofte, Hellerup, Denmark; 7 Department of Anaesthesiology, Zealand University Hospital, Koege, Denmark; 8 Department of Anaesthesiology and Intensive Care, Aarhus University Hospital, Aarhus, Denmark; 9 Department of Physiology and Pharmacology, Section of Anaesthesia and Intensive Care, Karolinska Institutet, Stockholm, Sweden; 10 Department of Perioperative Medicine and Intensive Care, Karolinska University Hospital, Stockholm, Sweden; 11 Department of Clinical Science and Education, Centre for Resuscitation Science, Karolinska Institutet, Södersjukhuset, Stockholm, Sweden; 12 Department of Surgery, Södersjukhuset, Stockholm, Sweden

**Keywords:** COVID-19, Glucocorticoids, Steroids, Intensive care, Thrombosis, Thromboembolism, Pulmonary embolism, Bleeding

## Abstract

**Background:**

Thromboembolism is more common in patients with critical COVID-19 than in other critically ill patients, and inflammation has been proposed as a possible mechanism. The aim of this study was to investigate if 12 mg vs. 6 mg dexamethasone daily reduced the composite outcome of death or thromboembolism in patients with critical COVID-19.

**Methods:**

Using additional data on thromboembolism and bleeding we did a post hoc analysis of Swedish and Danish intensive care unit patients enrolled in the blinded randomized COVID STEROID 2 trial comparing 12 mg vs. 6 mg dexamethasone daily for up to 10 days. The primary outcome was a composite outcome of death or thromboembolism during intensive care. Secondary outcomes were thromboembolism, major bleeding, and any bleeding during intensive care.

**Results:**

We included 357 patients. Whilst in intensive care, 53 patients (29%) in the 12 mg group and 53 patients (30%) in the 6 mg group met the primary outcome with an unadjusted absolute risk difference of − 0.5% (95% CI − 10 to 9.5%, p = 1.00) and an adjusted OR of 0.93 (CI 95% 0.58 to 1.49, p = 0.77). We found no firm evidence of differences in any of the secondary outcomes.

**Conclusions:**

Among patients with critical COVID-19, 12 mg vs. 6 mg dexamethasone daily did not result in a statistically significant difference in the composite outcome of death or thromboembolism. However, uncertainty remains due to the limited number of patients.

**Supplementary Information:**

The online version contains supplementary material available at 10.1186/s13613-023-01115-y.

## Background

Thromboembolism (TE) has been reported to be three times more common in patients with critical COVID-19 than in other critically ill patients [1, 2]. Inflammation has been proposed as a mechanism for TE in COVID-19 patients. Coagulopathies in COVID-19 differ from disseminated intravascular coagulopathy mainly by a preserved platelet count, normal or minimally prolonged prothrombin time, and TE being more common than bleeding [3, 4]. Hypercoagulation in COVID-19 likely has several mechanisms; both direct viral factors of the SARS-CoV-2 and the immunological response to the infection can trigger coagulation pathways [5–9]. In summary, the inflammatory state caused by COVID-19 may disrupt the anti-thrombotic and anti-inflammatory functions of endothelial cells causing both microvascular and macrovascular thrombosis [10, 11]. One of the first interventions to decrease the risk of TE in patients with critical COVID-19 was to use intensified thromboprophylaxis. However, more recent data suggests there is no survival benefit over the standard dose [12, 13].

In June 2020, the RECOVERY trial, comparing treatment with 6 mg dexamethasone vs. standard care without glucocorticoids, reported a 12% absolute risk reduction (risk ratio 0.64, 95% CI 0.51–0.81) in 28 day mortality with dexamethasone for patients requiring invasive respiratory support [14]. Since the publication of this trial, 6 mg dexamethasone daily has become standard treatment for patients with severe or critical COVID-19 [15, 16]. Additionally, the COVID STEROID 2 trial found high probabilities of better outcomes with 12 mg vs. 6 mg dexamethasone, although the pre-defined threshold for statistical significance was not met in the primary analysis [17, 18].

Whether treatment with higher doses of glucocorticoids can decrease the risk of TE in critically ill COVID-19 patients compared to lower doses is unknown. On one hand, the increased attenuation on inflammation could decrease coagulation triggers. On the other hand, glucocorticoids have also been suggested to increase the risk of TE which could be worse with a higher dose [19]. Also, higher doses of glucocorticoids may increase the risk of bleeding and thereby add to the risk of gastrointestinal (GI) bleeding in critically ill patients [20]. In more recent studies, the risk of GI bleeding has only been slightly increased or not confirmed at all and is all over low for critically ill patients [21]. Likewise, in the COVID STEROID 2 trial, the incidence of severe GI bleeding was very low in both the 12 and the 6 mg group, 1.8% and 1.0%, respectively [17, 22].

The aim of this post hoc study was to investigate if 12 mg vs. 6 mg dexamethasone daily reduced the composite outcome of death or thromboembolism in patients with critical COVID-19.

## Methods

This study is a post hoc analysis of the international, randomized, blinded COVID STEROID 2 trial investigating the subgroup of patients enrolled at ICUs in Denmark and Sweden [17]. The COVID STEROID 2 trial and collection of data to local databases were approved by medical agencies and ethics committees in Denmark (Ethics committee number H-20051056, 31-1521-293, R-21004283, EudraCT number 2020-003363-25, Danish Medicines Agency number 2020-07-16) and Sweden (Ethics committee number 2020-02582, 2020-04403, 2022-00152-022020-01302, 2020–02890, 2020-06674, EudraCT 2020-001395-15).

The enrollment of patients in the COVID STEROID 2 trial was done according to national regulations. In Denmark, informed consent was obtained from patients, a legal surrogate, or as an emergency procedure (by a physician not involved in the trial) before enrolling in the study. If a patient was enrolled by emergency procedure a consent was later obtained from the patient or legal surrogate to continue the participation. In Sweden informed consent was obtained from patients. If the patient withdrew consent, already collected data could be used as explicitly permitted with the initial consent.

This report was prepared in accordance with the STROBE checklist.

### Trial sites and patients

Between August 27, 2020, and May 20, 2021, 1000 patients were screened and enrolled in the COVID STEROID 2 trial; inclusion criteria were hospitalized patients ≥ 18 years of age with PCR-confirmed SARS-CoV-2 and treatment for hypoxia with at least 10 L/min of oxygen, non-invasive mechanical ventilation, continuous positive airway pressure, or invasive mechanical ventilation. Patients were excluded if previously randomized to the COVID STEROID 2 trial, if they had already received glucocorticoids for COVID-19 for more than four consecutive days, had treatment with glucocorticoids in doses higher than 6 mg dexamethasone for an indication other than COVID-19, had a diagnosis of active tuberculosis, or active fungal infection, had hypersensitivity to dexa-/betamethasone, or if they were pregnant.

The additional inclusion criteria for this post hoc analysis was to only include patients randomized in an ICU in hospitals in Denmark or Sweden as full data on TE and bleeding were only available in this group. The additional exclusion criteria were established TE and/or bleeding at randomization date. Patients were followed until ICU discharge, death, or withdrawal of consent.

### Randomization and intervention

Patients in the COVID STEROID 2 study were randomized 1:1 to 12 mg or 6 mg dexamethasone daily. At sites where dexamethasone was not available, betamethasone was permitted as the drugs are likely equipotent [23].

### Data collection

Local trial investigators entered data in the COVID STEROID 2 database using web-based case report files, including data regarding baseline characteristics, mortality and allocation. Data on TE and bleeding, laboratory coagulation parameters, and data on anticoagulation regime were retrieved from local databases. Both in Denmark and Sweden, these local databases were built by reviewing patient data in electronic health records (EHR). This was done by medical students, physicians, and research nurses. If the EHR was difficult to interpret, the reviewer was instructed to discuss with the responsible investigator (i.e., an experienced senior physician) at the site. Regimes of anticoagulation were categorized as follows: low dose of low-molecular-weight heparin (LMWH): 2500–4500 IU daily for tinzaparin, 2500–5000 IU daily for dalteparin or ≤ 40 mg daily for enoxaparin; intermediate dose of LMWH: > 4500 IU but < 175 IU/kg of body weight daily for tinzaparin, > 5000 IU but < 200 IU/kg of body weight daily for dalteparin, or > 40 mg but < 1 mg/kg of body weight daily for enoxaparin; and high dose of LMWH: ≥ 175 IU/kg of body weight daily for tinzaparin, ≥ 200 IU/kg of body weight daily for dalteparin, or ≥ 1 mg/kg of body weight daily of enoxaparin.

### Outcome

The primary outcome of this post hoc study was the composite outcome of death or TE during ICU stay. Secondary outcomes were TE, major bleeding, and any bleeding during ICU stay. TE was a composite of clinically detected myocardial infarction (MI), pulmonary embolism/thrombosis (PE/PT), deep vein thrombosis (DVT), ischemic stroke, or other thromboembolic events. Diagnoses were confirmed by computed tomography (for PE/PT, ischemic stroke, other thromboembolic events), ultrasound (DVT) or according to the fourth universal definition of myocardial infarction by European Society of Cardiology (MI) [24]. Bleeding was registered according to site and severity and categorized as major bleeding or any bleeding. Major bleeding was defined as a bleeding requiring transfusion of at least two units of packed red blood cells, intracranial bleeding, and/or a bleeding requiring a major therapeutic intervention, e.g., surgery or interventional radiology. Any bleeding was defined as a bleeding described in the EHR. No center preformed screening for TE or bleeding, and examinations were done at the discretion of the treating clinicians. For both primary and secondary outcomes, the interaction of baseline fibrin-D-dimer and CRP were investigated.

### Statistical analysis

All analyses were done by intention to treat. Descriptive statistics were used to summarise baseline and follow-up data with medians and interquartile range (IQR) presented for continuous data and numbers and proportions (%) for categorical data. A two-sample test for equality of proportions was used to estimate the unadjusted risk difference between groups. Regression analyses were used to assess the outcomes from randomization to ICU discharge, death in the ICU, or withdrawal of consent, whichever occurred first. Logistic regression was used to estimate the odds ratios (ORs) with corresponding 95% confidence intervals (CIs) for the primary and secondary outcomes. Cox proportional hazards regression was used to estimate hazard ratios (HRs) with corresponding 95% CIs for time to primary and secondary outcomes with patients right-censored when discharged from ICU, when dying in ICU, or withdrawing consent from the study. The regression analyses for both primary and secondary outcomes was performed with and without adjusting for age (below 70 years vs. 70 years or above) and the use of invasive mechanical ventilation at screening (yes/no) as in the COVID STEROID 2 trial. In addition, adjustment for initial dose of LMWH (low, intermediate, or high dose) in the ICU was also performed in an additional logistic regression analysis.

The fibrin-D-dimer and CRP and their interactions with the dose of dexamethasone were modeled using restricted cubic splines in separate logistic regression models. Reference intervals on fibrin-D-dimer differed between hospitals. All hospitals provided an absolute value if the result was between 0.2 and 12 mg/L fibrinogen equivalent units (FEU). Below 0.2 and above 12 mg/L FEU, the value could be noted as less than 0.2 and more than 12 mg/L FEU. This was handled by replacing values above 12 mg/L by 13 mg/L and below 0.2 mg/L by 0.19 mg/L FEU. This was deemed unproblematic since clinically values below 0.2 mg FEU are considered very low and above 12 mg FEU are considered very high and medians and IQR were used to present the result.

To check the assumption of proportional hazards, scaled Schoenfeld residuals were plotted and there was no indication of violation.

Two-sided p-values < 0.05 was considered statistically significant. SPSS Statistics v 28.0.0.0 (190) (IBM, 2021) and R v 3.5.1 (R Core, 2017. R: A language and environment for statistical computing. R Foundation for Statistical Computing, Vienna, Austria) were used for analysis.

## Results

### Study population

**Fig. 1:**
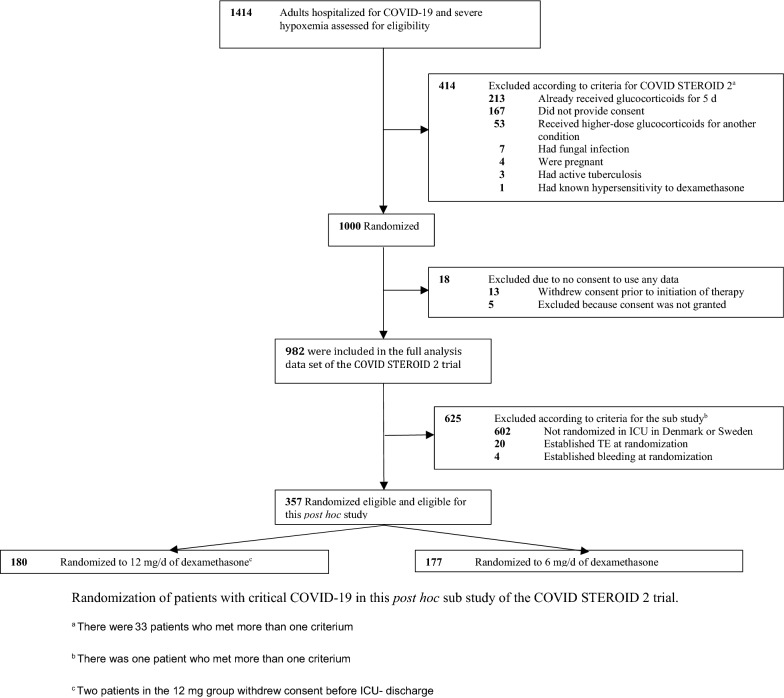
Patients with critical COVID-19 in the *post hoc,* study of the COVID STEROID 2 trial

Out of the 1000 patients randomized in the COVID STEROID trial, 380 patients were enrolled in a Danish or Swedish ICU. Nineteen patients were excluded due to TE, three due to bleeding and one due to both TE and bleeding at the time of randomization. Included patients were from 19 different sites, 17 in Denmark (300 patients) and two in Sweden (57 patients). All of the 357 patients, 180 allocated to the 12 mg group and 177 allocated to the 6 mg group, were followed until ICU-discharge, death, or withdrawal of consent (Fig. 1).

Baseline characteristics are presented in Table 1. The median time from ICU admission to randomization was 1 day (range 0–5), and patients were treated in the ICU for median 9 days (range 0–96).

**Table 1:** Baseline characteristics by dose of dexamethasone

Characteristic	12 mg of dexamethasone (n = 180)	6 mg of dexamethasone (n = 177)
Country of enrollment		
Denmark	152 (84)	148 (84)
Sweden	28 (16)	29 (16)
Age, median (IQR) years	64 (57 to 74)	66 (58 to 73)
Sex		
Male	121 (67)	122 (69)
Female	59 (33)	55 (31)
Weight, median (IQR) kg	90 (75 to 105)	90 (78 to 103)
Coexisting conditions		
Diabetes mellitus	34 (19)	53 (30)
Ischemic heart disease or heart failure	23 (13)	23 (13)
Chronic obstructive pulmonary disease	20 (11)	28 (16)
Immunosuppressive therapy within 3 months prior to randomization	13 (7.2)	10 (5.6)
Limitations of care (life support or CPR)	14 (7.8)	12 (6.8)
Time from onset of symptoms to hospitalization, median (IQR), days	(n = 163), 7 (4 to 10)	(n = 166), 8 (5 to 10)
Time from hospitalization to randomization, days	2 (1 to 3)	1 (1 to 3)
Time from hospitalization to ICU admission, days	1 (0 to 1)	1 (0 to 1)
Type of oxygen supplementation		
Nasal cannula or open mask	99 (55)	101 (57)
Flow rate for nasal cannula and open mask, median (IQR) L/min	25 (15 to 41)	29 (17 to 45)
Closed systems	81 (45)	76 (43)
Non-invasive ventilation	23 (13)	24 (14)
Continuous positive airway pressure	1 (0.6)	2 (1.1)
Invasive ventilation	57 (32)	50 (28)
PaO2/FiO2 for closed systems, median (IQR)	(n = 79), 17 (12 to 23)	(n = 76), 17 (11 to 22)
Chronic use of medication		
Corticosteroids	2 (1.1)	12 (6.8)
Antithrombotic medication	(n = 177), 51 (29)	(n = 172), 49 (28)
Therapy during current admission		
Anti-inflammatory agents	22 (12)	23 (13)
Janus Kinase inhibitor	0 (0)	0 (0)
IL-6 inhibitor	20 (11)	18 (10)
Other	3 (1.7)	5 (2.8)
Antiviral agents	99 (55)	94 (53)
Remdesivir	95 (53)	92 (52)
Convalescent plasma	1 (0.6)	2 (1.1)
Other	6 (3.3)	2 (1.1)
Antibacterial medication in the 24 h prior to randomization	107 (59)	116 (66)
Vasopressor or inotrope for at least 1 h in the 24 h prior to randomization	50 (28)	36 (20)
Renal replacement therapy in the last 72 h prior to randomization	5 (2.8)	2 (1.1)
Initial dose of LMWH^a^	(n = 167)	(n = 168)
High LMWH dose^b^	33 (20)	30 (18)
Intermediate LMWH dose^c^	86 (51)	88 (52)
Low LMWH dose^d^	45 (27)	48 (29)
No prophylaxis	3 (1.8)	2 (1.2)
Laboratory markers		
Plasma Lactate^e^, median (IQR) mmol/L	(n = 177), 1.8 (1.3 to 2.7)	1.9 (1.4 to 2.5)
Hemoglobine^a^, median (IQR) mmol/L	(n = 179), 8.1 (7.3 to 8.8)	(n = 176), 8.1 (7.2 to 8.7)
Platelet count^a^, median (IQR) 10^9/L	(n = 172) 234.0 (187.5 to 308.0)	(n = 175) 229.0 (176.0 to 301.5)
Prothrombin time^a^, median (IQR) INR	(n = 170), 1.1 (1.0 to 1.2)	(n = 169), 1.1 (1.0 to 1.2)
Fibrin-D-dimer^a^, median (IQR) mg FEU (< 0.2—> 12) mg/L	(n = 153), 1.1 (0.7 to 2.5)	(n = 152), 1.3 (0.9 to 2.1)
CRP^a^, median (IQR) mg/L	(n = 175), 120 (81 to 170)	(n = 175), 128 (82 to 199)

Baseline characteristics of 357 patients with critical COVID-19 by dose of dexamethasone. Values are expressed as no. (%) unless otherwise indicated. Data are complete for all included patients unless indicated by number of patients. For proportions of missing data se additional file, handling of missing data.

*CPR* cardiopulmonary resuscitation, *PaO2/FiO2* Partial pressure of oxygen/Fraction of inspired oxygen, *IL-6* Interleukin-6, *LMWH* low-molecular-weight heparin, *ICU* intensive care unit, *FEU* fibrinogen equivalent units, *CRP* c-reactive protein

^a^At ICU admission defined as the first date during ICU stay

^b^Tinzaparin, ≥ 175 IU/kg of body weight per daily, dalteparin, ≥ 200 IU/kg of body weight daily, or enoxaparin, ≥ 1 mg/kg of body weight daily

^c^Tinzaparin, > 4500 IU daily to < 175 IU/kg of body weight daily, or dalteparin, > 5000 IU daily to < 200 IU/kg of body weight daily, or enoxaparin, > 40 mg but < 1 mg/kg of body weight daily

^d^Tinzaparin, 2500–4500 IU daily, dalteparin, 2500–5000 IU daily, or enoxaparin, ≤ 40 mg daily

^e^SI conversion factor: to convert lactate to mg/dL divide by 0.111

### Primary outcome

**Fig. 2:**
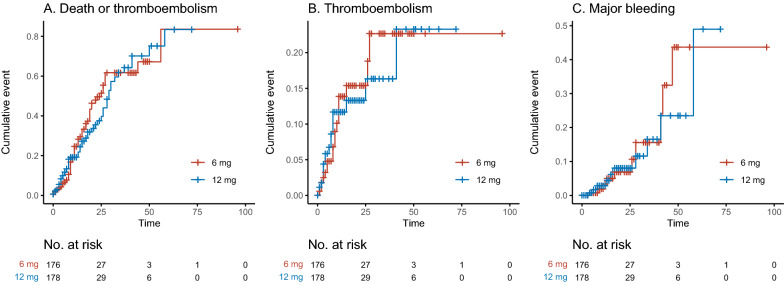
Kaplan–Meier curves of outcomes during ICU stay according to dose of dexamethasone. Kaplan–Meier curves of **A** Death or Thromboembolism **B** Thromboembolism, and **C** Major bleeding events among 354 patients with critical COVID-19 by dose of dexamethasone. Thromboembolism in **A** and **B** defined as myocardial infarction, pulmonary embolism/thrombosis, deep vein thrombosis, ischemic stroke or other thromboembolic events. Major bleeding in **C** defined as a bleeding requiring transfusion of at least two units of red blood cells, an intracranial bleeding, and or a bleeding requiring a major therapeutic intervention

During ICU stay, 53 patients (29%) in the 12 mg group and 53 patients (30%) in the 6 mg group met the primary outcome of death or TE, absolute risk difference between groups 0.5% (95% CI − 9.5 to 10%, p = 1.00, Table 2). The cumulative proportions of death or TE were similar between groups (Fig. 2a). The adjusted OR for the primary outcome for the 12 mg vs. 6 mg group was 0.93 (95% CI 0.58 to 1.49, p = 0.768, Table 3). The result was similar after additional adjustment for initial LMWH-dose (Additional file 2: Table S1). The adjusted HR for primary outcome for the 12 mg vs. 6 mg group was 0.95 (CI 95% 0.65 to 1.40, p = 0.813) (Additional file 2: Table S2).

**Table 2:** Primary and secondary outcomes by dose of dexamethasone

Outcome	12 mg of dexamethasone (n = 180)	6 mg of dexamethasone (n = 177)	Absolute differences (%)	95% CI (%)	p-value^a^
Death or thromboembolism	53 (29)	53 (30)	− 0.50	− 10 to 9.5	1.00
Death	40 (22)	40 (23)	− 0.38	− 9.4 to 8.7	1.00
Thromboembolism	18 (10)	18 (10)	− 0.17	− 6.6 to 6.2	1.00
Pulmonary embolism	14 (7.8)	17 (9.6)	− 1.8	− 8.2 to 4.6	0.67
Deep vein thrombosis	0 (0)	0 (0)	0.00		
Myocardial infarction	2 (1.1)	0 (0)	1.1	− 1.0 to 3.2	0.49
Stroke	1 (0.6)	0 (0)	0.56	− 1.1 to 2.2	1.00
Other thrombotic event	3 (1.7)	3 (1.7)	− 0.03	− 2.7 to 2.7	1.00
Type of bleeding					
Major bleeding	10 (5.6)	10 (5.6)	− 0.09	− 5.0 to 4.8	1.00
Any bleeding	35 (19)	41 (23)	− 3.7	− 13 to 5.3	0.47
Site of bleeding					
Upper airway bleeding	13 (7.2)	16 (9.0)	− 1.8	− 8.0 to 4.4	0.66
Lower airway bleeding	4 (2.2)	6 (3.4)	− 1.2	− 5.2 to 2.8	0.73
Intrathoracic airway bleeding (hemothorax, mediastinum, pleura),	0 (0)	3 (1.7)	− 1.7	− 4.2 to 0.77	0.24
Skin (petechiae, ecchymosis, purpura),	4 (2.2)	3 (1.7)	0.53	− 2.9 to 3.9	1.00
IV lines: bleeding from catheters, drains, iv lines	9 (5.0)	8 (4.5)	0.48	− 4.4 to 5.4	1.00
Muscle: bleeding in muscle or soft tissue,	5 (2.8)	4 (2.3)	0.52	− 3.2 to 4.3	1.00
Upper GI bleeding	9 (5.0)	9 (5.1)	− 0.08	− 4.7 to 4.5	1.00
Lower GI bleeding	5 (2.8)	2 (1.1)	1.6	− 1.8 to 5.1	0.46
Intracranial bleeding	1 (0.6)	1 (0.6)	− 0.01	− 1.6 to 1.5	1.00
Post-surgical bleeding	5 (2.8)	4 (2.3)	0.52	− 3.2 to 4.3	1.00
Genitourinary bleeding	5 (2.8)	5 (2.8)	− 0.05	− 3.5 to 3.4	1.00

Primary and secondary outcomes during ICU stay among 357 patients with critical COVID-19 by dose of dexamethasone. Values are expressed as no. (%)

*CI* confidence interval, *GI* gastrointestinal

^a^Two sample test for equality of proportions; 2-sample test for equality of proportions without continuity correction

**Table 3:** Risk of death or thromboembolism, thromboembolism, major bleeding, and any bleeding by dose of dexamethasone

Characteristic	OR	95% CI	p-value
Death or thromboembolism, unadjusted	0.98	0.62 to 1.54	0.92
Death or thromboembolism, adjusted^a^	0.93	0.58 to 1.49	0.77
Thromboembolism, unadjusted	0.98	0.49 to 1.96	0.96
Thromboembolism, adjusted^a^	0.97	0.48 to 1.94	0.93
Major bleeding, unadjusted	0.98	0.39 to 2.45	0.97
Major bleeding, adjusted^a^	0.97	0.39 to 2.42	0.94
Any bleeding, unadjusted	0.80	0.48 to 1.33	0.39
Any bleeding, adjusted^a^	0.78	0.46 to 1.30	0.34

Odds ratios for death or thromboembolism, thromboembolism, major bleeding and any bleeding during ICU stay among 357 patients with critical COVID-19 with 12 mg vs. 6 mg dexamethasone daily

*OR* odds ratio, *CI* confidence interval

^a^Adjusted for age (< / ≥ 70 years) and invasive mechanical ventilation (yes/no)

### Secondary outcome

No firm evidence of differences was found in any of the secondary outcomes (TE, major bleeding, or any bleeding) between the 12 and 6 mg group (Tables 2, 3). Eighteen patients (10%) in the 12 mg group and 18 patients (10%) in the 6 mg group had TE (absolute risk difference 0.17%; 95% CI − 6.2 to 6.6%, p = 1.00; Table 2). Cumulative proportions are displayed in Fig. 2b. There were no statistically significant differences between the different types of TEs with the majority being PE/PT in both groups (Table 2). The adjusted OR for TE for the 12 mg was 0.97 (95% CI 0.48 to 1.94, p = 0.927) vs. the 6 mg group (Table 3), and the adjusted HR for TE for the 12 mg was 1.01 (CI 95% 0.52 to 1.93, p = 0.988) vs. the 6 mg group (Additional file 2: Table S2).

Bleeding occurred in 19% (35/180) in the 12 mg group and 23% (41/177) in the 6 mg group leading to a risk difference of 3.7% (95% CI − 5.3% to 13%, p = 0.446); in both groups 5.6% had major bleeding. The adjusted ORs for bleeding and major bleeding were 0.80 (95% CI 0.46 to 1.30, p = 0.339) and 0.97 (95% CI 0.39 to 2.42, p = 0.940), respectively (Table 3). The adjusted HR for major bleeding was 0.89 (95% CI 0.36 to 2.15, p = 0.789) for the 12 mg group vs. the 6 mg group as seen in Additional file 2: Table S2; the cumulative proportions are shown in Fig. 2c. The bleeding sites did not differ substantially between groups (upper airway bleeding in 9.0% and 7.2% of patients, upper GI bleeding in 5.1% and 5.0%, and bleeding from intravenous lines in 4.5% and 5.0% in the 12 mg and 6 mg groups, respectively, Table 2).

### LMWH, Fibrin-D-dimer, and CRP

There were considerable uncertainties in the analyses when separately analyzing patients with high, intermediate, and low dosing of LMWH against dose of dexamethasone (Additional file 2: Table S3).

Interactions between the intervention and both fibrin-D-dimer and CRP were investigated for all outcomes with no statistically significant different result and substantial uncertainty in the results (Additional file 2: Fig S1).

## Discussion

In this post hoc analysis of the COVID STEROID 2 trial, we found no firm evidence of differences in risk of the composite outcome of death or TE, TE, major bleeding, or any bleeding during ICU stay for patients with critical COVID-19 receiving 12 mg vs. 6 mg dexamethasone. The multicenter randomized COVIDICUS trial, comparing high-dose glucocorticoids (dexamethasone, 20 mg day 1–5 and 10 mg day 6–10) with standard dose, reported on the safety outcomes of TE and bleedings [25]. In contrast to our study, COVIDICUS was constrained to PE, DVT, and GI bleedings. Nevertheless, results are consistent with ours, although both studies are limited by the low number of events.

One possible reason for not finding a difference in death, thromboembolism or bleeding between 12 and 6 mg group could be that coagulopathic side-effects of glucocorticoids counteract the glucocorticoids’ attenuation on inflammatory induced TE. In a meta-analysis of retrospective and observational studies with COVID-19 patients, glucocorticoids increased the incidence of TE, but the study did not consider ICU patients as a separate cohort [19]. The side effects of glucocorticoids are well known as glucocorticoids are used in the treatment of many diseases. Long term use of glucocorticoids is associated with coagulopathy, both venous thromboembolism and bleeding, mainly from the GI tract [26]. Lately, short-term use of glucocorticoids has been identified to increase risk of coagulopathy, especially within 30 days of treatment [27]. Consequently, an equilibrium between glucocorticoid effect on inflammatory induced TE and glucocorticoid side effects with increased doses of dexamethasone from 6 to 12 mg daily could result in a similar incidence of TE and bleeding in the two groups.

As inflammation has been proposed as a mechanism for TE, we wanted to investigate if patients with a higher degree of inflammation had a different outcome according to the dose of dexamethasone compared to the patients with a lower degree. For this, we used Fibrin-D-dimer as it is an indicator of clotting disorder and extensively studied in the COVID-19 population, and CRP, a well-established marker of inflammation. Analyses of possible interactions between the dose of dexamethasone and Fibrin-D-dimer and CRP revealed no significant results. As visualized in Additional file 2: Fig S1a–c, the point estimate was lower for death and TE, TE, and major bleeding in the 12 mg group with a baseline CRP between 80 and 250, although these results carry substantial uncertainties.

Treatment guidelines have evolved rapidly during the pandemic, and it is difficult to separate the introduction of treatment with glucocorticoids from the effect of other temporal changes in treatment with intensified thromboprophylaxis being one important confounder [3]. In our study, we additionally adjusted for different doses of LMWH and looked at each LMWH subgroup separately. However, the outcomes did not differ significantly within LMWH-doses between the 12 and the 6 mg group.

### Limitations

Our post hoc analysis has several limitations. First, the absence of a significant difference between groups may be due to limited power and therefore a substantial difference in both directions cannot be rejected. When planning for the study, we expected that all COVID STEROID 2 patients admitted to ICUs in Denmark and Sweden would be eligible, but some were randomized before ICU admission. To have access to full data of TE and bleeding events, we only included patients randomized after ICU admission. However, because the outcome of TE in our population was quite rare, the possibility to show a difference between the groups would require a sample size so large that the effect of the intervention would most likely not be clinically meaningful.

Second, in the additional analyses including daily dose of LMWH, we grouped patients according to the initial dose when admitted to the ICU. This could potentially be a misclassification as many patients change LMWH dose during the ICU-stay. For us this was the only availably way as the Swedish patients only had initial dose registered. The results of the analysis including LMWH must only be seen as an attempt to illustrate thromboprophylaxis as it is of interest in a study with outcomes of thromboembolism and bleeding. However, as the patients were randomized to dose of dexamethasone the possible misclassification is random and should not produce systematic difference in the risk of changes of LMWH dose between the 12 vs. 6 mg groups.

Third, date of ICU admission and randomization could be on different dates. This adds to the risk of misclassification of LMWH dose. This was also the case when investigating interactions with Fibrin-D-dimer and CRP as the available laboratory results are from the ICU admission date. However, the median difference between ICU-admission and randomization was only one day.

Fourth, we choose discharge from ICU as the follow up period of our primary endpoint. An alternative follow-up period would have been a fixed period, for example 28 days from ICU admission. The reason for our choice was the post hoc nature of the study and the consequent limited availability of data.

Finally, the diagnosis of TE may have been missed as the incidence was low compared to that reported in a meta- analysis of TE in COVID-19 patients [28]. In our population, the incidence of TE was only 10% in both groups with especially low numbers for DVT and arterial thromboembolic events. However, this low prevalence is in concordance with newer RCTs [29, 30]. One reason could be that our patients were not screened for TE. Guidelines recommend a low threshold for proceeding with an investigation for TE but yet screening for all patients is not endorsed and the diagnosis may be missed [31]. Studies using routine screening of TE show higher incidence compared to those relying on clinical suspicion [28, 32, 33]. This indicates that for future studies investigating TE in patients with critical COVID-19, routine screening will likely find more TE and therefore may also find difference between interventions. However, the clinical importance of asymptomatic TE must be considered. Other reasons for the low incidence of TE could be the exclusion of patients with TE diagnosed at ICU admission and the use of intensified thromboprophylaxis; high or intermediate dose of LMWH as starting dose were prescribed to most of the patients in our study.

## Conclusions

Among patients with critical COVID-19, 12 mg vs. 6 mg of dexamethasone daily did not result in a statistically significant difference in the composite outcome of death or thromboembolism**.** However, uncertainty remains due to the limited number of patients.

## Supplementary Information

**Additional file 1: **STROBE Statement—checklist of items that should be included in reports of observational studies

**Additional file 2: Figure S1.** Outcome by laboratory markers as interaction with dose of dexamethasone, **Table S1.** Risk of death or thromboembolism, thromboembolism, major bleeding and any bleeding by dose of dexamethasone adjusting for dose of LMWH, **Table S2.** Adjusted risk of death or thromboembolism, thromboembolism and major bleeding by dose of dexamethasone, **Table S3.** Incidence of death or thromboembolism, thromboembolism, major bleeding and any bleeding by dose of dexamethasone and dose of LMWH.

## Data Availability

The data sets generated and analyzed during the current study are not publicly available due to patient records’ regulations but can be made available by corresponding author on reasonable request.

## Abbreviations

ARDS: Acute respiratory distress syndrome
CI: Confidence interval
CRP: C-reactive protein
DVT: Deep vein thrombosis
EHR: Electronic health record
FEU: Fibrinogen equivalent units
GI: Gastrointestinal
HR: Hazard ratio
ICU: Intensive care unit
IQR: Interquartile range
KM: Kaplan-Meier
LMWH: Low-molecular-weight heparin
MI: Myocardial infarction
OR: Odds ratio
PE: Pulmonary embolism
PT: Pulmonary thrombosis
TE: Thromboembolism
