# Supplementary material for: Effects of 12 mg vs. 6 mg dexamethasone on thromboembolism and bleeding in patients with critical COVID-19 - a post hoc analysis of the randomized, blinded COVID STEROID 2 trial
Source: Ann Intensive Care. 2023 Mar 2;13:12. doi: 10.1186/s13613-023-01115-y (PMC9979892; doi:10.1186/s13613-023-01115-y)
Supplement: Supplementary file 2 — Additional file 2: Figure S1. Outcome by laboratory markers as interaction with dose of dexamethasone, Table S1. Risk of death or thromboembolism, thromboembolism, major bleeding and any bleeding by dose of dexamethasone adjusting for dose of LMWH, Table S2. Adjusted risk of death or thromboembolism, thromboembolism and major bleeding by dose of dexamethasone, Table S3. Incidence of death or thromboembolism, thromboembolism, major bleeding and any bleeding by dose of dexamethasone and dose of LMWH. [file 13613_2023_1115_MOESM2_ESM.docx]

# The effects of 12 mg vs. 6 mg dexamethasone on thromboembolism and bleeding in patients with critical COVID-19 - a *post hoc* analysis of the randomized, blinded COVID STEROID 2 trial

# Supplement

**Contents**

**Handling of missing data.** Complete missingness of data, missing baseline variables and missing outcome data

**Figure S1.** Outcome by laboratory markers as interaction with dose of dexamethasone

**Table S1.** Risk of death or thromboembolism, thromboembolism, major bleeding and any bleeding by dose of dexamethasone adjusting for dose of LMWH

**Table S2.** Adjusted risk of death or thromboembolism, thromboembolism and major bleeding by dose of dexamethasone

**Table S3.** Incidence of death or thromboembolism, thromboembolism, major bleeding and any bleeding by dose of dexamethasone and dose of LMWH

**Handling missing data in this *post hoc* study of the COVID STEROID 2 trial**

We did no imputation of missing data. Two patients in the 12 mg group withdrew consent at Day 2 and 4, respectively, but data up until withdrawal were analyzed.

*Complete missingness of data*

We had no patients with complete missingness of data.

*Missing baseline variables*

We had no data regarding onset of symptoms for 17 patients (9.4%) in the 12 mg group and eleven patients (6.2%) in the 6 mg group. Therefore, we could not calculate days from onset to hospital admission. All patients had a measurement of oxygen support but for open systems this was indicated by liter of oxygen per minute and for closed systems as fraction of inspired oxygen. Data on chronic use of antithrombotic therapy was missing for three patients (1.7%) in the 12 mg group and five patients (2.8%) in the 6 mg group. Dose of low-molecular-weight heparin at ICU admission were missing for 13 patients (7.2%) in the 12 mg group and nine patients (5.1%) in the 6 mg group. For laboratory data at ICU admission hemoglobin were missing for one patients (0.6%) in the 12 mg group and one patient (0.6%) in the 6 mg group, platelets were missing for eight patients (4.4%) in the 12 mg group and two patients (1.1%) in the 6 mg group, prothrombin time were missing for ten patients (5.6%) in the 12 mg group and eight patients (4.5%) in the 6 mg group, fibrin-D-dimer were missing for 27 patients (15.0%) in the 12 mg group 25 patients (14.1%) in the 6 mg group and C-reactive protein were missing for five patients (2.8%) in the 12 mg group and two patients (1.1%) in the 6 mg group.

*Missing outcome data*

For the primary outcome analyzed with logistic regression we had no missing data. When analyzing the primary outcome using Cox regression one patient in the 6 mg group and two patients in the 12 mg group were excluded due to missing date of ICU-discharge.

**Figure S1.** Outcome by laboratory markers as interaction with dose of dexamethasone


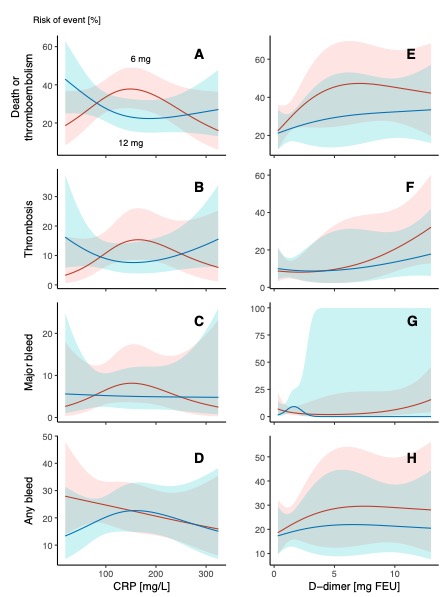
**Figure S1. Outcome by laboratory markers as interaction with dose of dexamethasone**

Outcome by laboratory markers of Fibrin-D-dimer and C-reactive protein at ICU admission when including interactions of Fibrin-D-dimer and C-reactive protein with the dose of dexamethasone. Blue line indicating risk for 12 mg group and red line indicating risk for 6 mg group. Percentage of patients with outcome on y-axis.

**A** Death or Thromboembolism by Fibrin-D-dimer including interaction with dose of dexamethasone **B** Thromboembolism by Fibrin-D-dimer including interaction with dose of dexamethasone **C** Major bleeding by Fibrin-D-dimer including interaction with dose of dexamethasone **D** Any bleeding by Fibrin-D-dimer including interaction with dose of dexamethasone **E** Death or Thromboembolism by C-reactive protein including interaction with dose of dexamethasone **F** Thromboembolism by C-reactive protein including interaction with dose of dexamethasone **G** Major bleeding by C-reactive protein including interaction with dose of dexamethasone **H** Any bleeding by C-reactive protein including interaction with dose of dexamethasone

**Table S1.**

Risk of death or thromboembolism, thromboembolism, major bleeding and any bleeding by dose of dexamethasone adjusting for dose of LMWH^a^

| Characteristic | OR | 95% CI | p-value |
| --- | --- | --- | --- |
| Death or thromboembolism, adjusted^b^ | 0.87 | 0.53 to 1.42 | .58 |
| Thromboembolism, adjusted^b^ | 1.00 | 0.48 to 2.05 | .99 |
| Major bleeding, adjusted^b^ | 0.74 | 0.26 to 2.06 | .57 |
| Any bleeding, adjusted^b^ | 0.73 | 0.42 to 1.26 | .26 |

Odds ratios for of death or thromboembolism, thromboembolism, major bleeding and any bleeding during ICU stay among 330 patients with critical COVID-19 with 12 mg vs. 6 mg dexamethasone daily.

Abbreviations: OR, Odds Ratio, CI, confidence interval, LMWH, low-molecular-weight heparin.

^a^ Excluding patients without thromboprophylaxis with LMWH (two in 6 mg group and three in 12 mg group) and when dose of LMWH was missing (nine in 6 mg group and 13 for 12 mg group).

^b^ Adjusted for age (</ ≥ 70 years), invasive mechanical ventilation (yes/no) and dose of LMWH (high/intermediate/low).

^c^ Tinzaparin, ≥ 175 IU/kg of body weight per daily, dalteparin, ≥ 200 IU/kg of body weight daily, or enoxaparin, ≥ 1 mg/kg of body weight daily.
^d^ Tinzaparin, > 4500 IU daily to < 175 IU/kg of body weight daily, or dalteparin, > 5000 IU daily to < 200 IU/kg of body weight daily, or enoxaparin, > 40 mg but < 1 mg/kg of body weight daily.
^e^ Tinzaparin, 2500–4500 IU daily, dalteparin, 2500–5000 IU daily, or enoxaparin, ≤ 40 mg daily.

**Table S2.**

Adjusted risk of death or thromboembolism, thromboembolism, and major bleeding by dose of dexamethasone^a^

| Characteristic | HR | 95% CI | p-value |
| --- | --- | --- | --- |
| Death or thromboembolism, adjusted^b^ | 0.95 | 0.65 to 1.40 | .81 |
| Thromboembolism, adjusted^b^ | 1.00 | 0.52 to 1.93 | .99 |
| Major bleeding, adjusted^b^ | 0.89 | 0.36 to 2.15 | .79 |

Hazard ratios for death or thromboembolism, thromboembolism and major bleeding during ICU stay among 354^c^ patients with critical COVID-19 with 12 mg vs. 6 mg dexamethasone daily.

Abbreviations: HR, hazard ratio, CI, confidence interval, ICU, intensive care unit.

^a^ Excluding patients with missing data for ICU-discharge (one in 6 mg group and two in 12 mg group).

^b^ Adjusted for age (</ ≥ 70 years) and invasive mechanical ventilation (yes/no).

^c^ Two patients in the 12 mg group censured not due to primary outcome but withdrawal of consent on day 2 and day 4, respectively.

**Table S3.**

Incidence of death or thromboembolism, thromboembolism, major bleeding, and any bleeding by dose of dexamethasone and LMWH^a^

| Outcomes^b^ | 12 mg of dexamethasone (n = 164) | 6 mg of dexamethasone (n = 166) | Absolute differences | 95 % CI | p-value^b^ |
| --- | --- | --- | --- | --- | --- |
| Thromboembolism or death |  |  |  |  |  |
| High dose LMWH^c^ | 9/33 (27.3%) | 10/30 (33.3%) | -6% | -28.8 to 16.6 | .78 |
| Intermediate dose LMWH^d^ | 23/86 (26.7%) | 22/88 (25.0%) | 1.7% | -11.2 to 14.8 | .86 |
| Low dose LMWH^e^ | 14/45 (31.1%) | 18/48 (37.5%) | -6.4% | -25.5 to 12.9 | .66 |
| Thromboembolism |  |  |  |  |  |
| High dose LMWH^c^ | 4/33 (12.1%) | 5/30 (16.7%) | -4.6% | -21.9 to 12.8 | .73 |
| Intermediate dose LMWH^d^ | 6/86 (7.0%) | 6/88 (6.8%) | 0.2% | -7.4 to 7.7 | 1.00 |
| Low dose LMWH^e^ | 7/45 (15.6%) | 6/48 (12.5%) | 3.1% | -11.1 to 17.2 | .77 |
| Major bleeding |  |  |  |  |  |
| High dose LMWH^c^ | 0/33 (0.0%) | 2/30 (6.7%) | -6.7% | -15.6 to 2.3 | .22 |
| Intermediate dose LMWH^d^ | 4/86 (4.7%) | 4/88 (4.6%) | 0.1% | -6.1 to 6.3 | 1.00 |
| Low dose LMWH^e^ | 3/45 (6.7%) | 3/48 (6.3%) | 0.4% | -9.6 to 10.4 | 1.00 |
| Any bleeding |  |  |  |  |  |
| High dose LMWH^c^ | 8/33 (24.2%) | 3/30 (10.0%) | 14.2% | -3.9 to 32.4 | .19 |
| Intermediate dose LMWH^d^ | 14/86 (16.3%) | 20/88 (22.7%) | -6.4% | -18.2 to 5.3 | .34 |
| Low dose LMWH^e^ | 8/45 (17.8%) | 15/48 (31.3%) | -13.5% | -30.7 to 3.8 | .15 |

Risk of death or thromboembolism, thromboembolism, major bleeding and any bleeding during ICU stay among 330 patients with critical COVID-19 by dose of dexamethasone and by dose of LMWH.

Values are expressed as No. (%).

Abbreviation: LMWH, low-molecular-weight heparin, CI, confidence interval.

^a^ Excluding patients without thromboprophylaxis with LMWH (two in 6 mg group and three in 12 mg group) and when dose of LMWH was missing (nine in 6 mg group and 13 for 12 mg group).

^b^ P-values for differences across exposure categories were obtained using two sample test for equality of proportions; 2-sample test for equality of proportions

^c^ Tinzaparin, ≥ 175 IU/kg of body weight per daily, dalteparin, ≥ 200 IU/kg of body weight daily, or enoxaparin, ≥ 1 mg/kg of body weight daily.
^d^ Tinzaparin, > 4500 IU daily to < 175 IU/kg of body weight daily, or dalteparin, > 5000 IU daily to < 200 IU/kg of body weight daily, or enoxaparin, > 40 mg but < 1 mg/kg of body weight daily.
^e^ Tinzaparin, 2500–4500 IU daily, dalteparin, 2500–5000 IU daily, or enoxaparin, ≤ 40 mg daily.
